# Genetic loci simultaneously controlling lignin monomers and biomass digestibility of rice straw

**DOI:** 10.1038/s41598-018-21741-y

**Published:** 2018-02-26

**Authors:** Zhen Hu, Guifen Zhang, Ali Muhammad, Rana Abdul Samad, Youmei Wang, Jonathan D. Walton, Yuqing He, Liangcai Peng, Lingqiang Wang

**Affiliations:** 1 Biomass and Bioenergy Research Centre, Huazhong Agricultural University, Wuhan, China; 2 National Key Laboratory of Crop Genetic Improvement, Huazhong Agricultural University, Wuhan, China; 3 College of Plant Science and Technology, Huazhong Agricultural University, Wuhan, China; 4 Department of Energy Plant Research Laboratory and DOE Great Lakes Bioenergy Research Center, Michigan State University, East Lansing, MI 48824 USA; 5 College of Life Science and Technology, Huazhong Agricultural University, Wuhan, China

## Abstract

Lignin content and composition are crucial factors affecting biomass digestibility. Exploring the genetic loci simultaneously affecting lignin-relevant traits and biomass digestibility is a precondition for lignin genetic manipulation towards energy crop breeding. In this study, a high-throughput platform was employed to assay the lignin content, lignin composition and biomass enzymatic digestibility of a rice recombinant inbred line population. Correlation analysis indicated that the absolute content of lignin monomers rather than lignin content had negative effects on biomass saccharification, whereas the relative content of *p*-hydroxyphenyl unit and the molar ratio of *p*-hydroxyphenyl unit to guaiacyl unit exhibited positive roles. Eight QTL clusters were identified and four of them affecting both lignin composition and biomass digestibility. The additive effects of clustered QTL revealed consistent relationships between lignin-relevant traits and biomass digestibility. Pyramiding rice lines containing the above four positive alleles for increasing biomass digestibility were selected and showed comparable lignin content, decreased syringyl or guaiacyl unit and increased molar percentage of *p*-hydroxyphenyl unit, the molar ratio of *p*-hydroxyphenyl unit to guaiacyl unit and sugar releases. More importantly, the lodging resistance and eating/cooking quality of pyramiding lines were not sacrificed, indicating the QTL information could be applied to select desirable energy rice lines.

## Introduction

Lignocellulosic biomass represents an abundant, renewable source of mixed sugars for fermentation to biofuels^1^. However, biomass feedstock conversion is currently a costly process because of the requirements for energetic pretreatment and expensive enzyme inputs to overcome biomass recalcitrance. One approach to overcome cell-wall recalcitrance is to develop bioenergy feedstock plants that are more susceptible to hydrolysis^2^. For this breeding purpose, it is necessary to uncover relationships between cell wall components, identify key cell wall compositional features influencing recalcitrance and discover and manipulate genetic basis involved in biosynthesis, modification and conversion of cell wall^3^.

Lignin is a hydrophobic heteropolymer composed of three types of hydroxycinnamyl alcohol precursors: syringyl (S), guaiacyl (G) and *p*-hydroxyphenyl (H). It has been identified as a major deterrent to enzyme attack on cellulose^4,5^. Lignin binds preferentially to the hydrophobic faces of cellulose but also to the cellulose-binding site of cellulases and is, therefore, a competitor in the interaction between cellulose and cellulases^6^. The accessibility of nonlignified cell walls to carbohydrate-binding modules (CBMs) showed a significantly negative correlation with lignin content^7^. Several studies also showed that improved biomass digestibility could be achieved by lignin removal^8,9^. However, lignin may not always be the dominant negative factor for saccharification efficiency. *Brachypodium distachyon sac1* and *sac2* mutants showed higher saccharification but no significant differences in lignin content compared to the wild type^2^. Six sorghum *brown midrib (bmr)* mutants showed reduced lignin concentration but a similar amount of glucose release to that observed in the wild type^10^. In a maize recombinant inbred population, the QTL controlling saccharification rates were independent of those for lignin abundance^11^.

In several studies, lignin monomer composition appeared much more important than lignin content for biomass conversion. The *Arabidopsis med5a/5b ref8* line with a similar lignin amount and almost exclusively *p*-hydroxyphenyl lignin subunits exhibited substantially facilitated polysaccharide saccharification^12^. The inconsistent roles of S/G in biomass digestion have been well summarized previously^4^. In addition, a few recent studies have reported the positive influence of H and H/G on biomass digestion. Density functional theory (DFT) calculations showed that the cleavage of β-O-4 linkages in the H-lignin dominant mutant was greater than in G-lignin dominant mutants^4^. In wheat accessions and rice mutants, H/G in KOH-extractable lignin showed a positive correlation with biomass digestibility^13^. In general, more studies are required to better define the impact of lignin content and composition on biomass recalcitrance.

Lignin-relevant traits and biomass digestibility are typically quantitatively inherited. The complex phenylpropanoid pathway leading to canonical monolignols was thought to be defined over a decade ago, but is still far from being fully understood. New enzymes representing new branches and/or the central pathway were recently discovered^14,15^. Mutagenesis-based single-gene cloning and candidate gene manipulation approaches for discovering new genes or regulators in the phenylpropanoid pathway are limited in the number of mutants and mutants often have unfavorable phenotypes^16–18^. QTL mapping, the process of constructing linkage maps and identifying genomic regions associated with traits, is a powerful approach for the dissection of complex traits and the cloning of corresponding genes. Moreover, DNA markers that are tightly linked to QTL and genes can be used as efficient molecular tools for marker-assisted selection (MAS) in plant breeding^19^. A number of QTL affecting grain yield and grain quality have been characterized in many crop plants^20,21^. However, studies focusing on detection of QTL associated with lignin-relevant traits or biomass digestibility are very limited. The QTL and candidate genes associated with lignin content and enzymatic digestibility were identified in a recombinant inbred maize population^11^. A total of 52 QTL for biomass compositional and bioconversion characters were detected in a forage maize doubled haploid (DH) population^22^. Further work should be conducted to detect more QTL related to lignin composition and biomass digestibility.

While QTL studies have the potential for the discovery of genes relevant to bioenergy, such an approach requires a robust and reliable high-throughput assay for large-scale screening of both cell wall composition and biomass digestibility. Until recently, several automated assay platforms have been reported to have sufficient sensitivity and reliability to undertake the necessary screening of large populations of plants for mutant identification and genome-wide genetic association studies^23–25^.

Rice is a major food crop and a model plant for cereal crops and biomass grasses. Using a high-throughput platform, we analyzed the lignin content, lignin composition and biomass bioconversion characteristics of the stem materials from a rice recombinant inbred line (RIL) population and then mapped the corresponding QTL. The correlations within/between lignin-relevant traits and biomass digestibility were evaluated by correlation analysis and QTL co-location information. Through marker-assisted selection, we selected several rice lines with modified lignin composition and improved sugar release. Interestingly, the lodging resistance, grain shape, weight and eating/cooking quality of pyramiding rice lines were not sacrificed.

## Results

### Diverse lignin monomers and biomass enzymatic digestibility in rice population

Using a high-throughput platform described in the methods section, we analyzed lignin content, lignin composition (n = 99) and biomass enzymatic digestibility (n = 215) of the rice straw harvested from a recombinant inbred line (RIL) population at the mature stage on a large scale (Table 1). The content of acetyl bromide soluble lignin ranged from 14.63 to 18.21% in isolated cell walls. For lignin monomers, guaiacyl unit (G) represented 49.95 to 64.4% of the total units, followed by the syringyl unit (S, 26.71 to 38.33%) and the *p*-hydroxyphenyl unit (H, 6.22 to 14.48%). The xylose (Xyl-Rel) and glucose (Glc-Rel) released after enzymatic digestion following mild NaOH pretreatment were approximately 4.06% and 14.59% of the dry biomass, respectively (Table 1).

**Table 1:** Statistic and diversity of lignin-relevant traits and enzymatic digestibility of the rice recombinant inbred line population and two parents.

Traits	Parents			Population				
	HH-3	ZX	*P* ^a^	Mean ± SD^b^	Min	Max	CV^c^	*P* ^d^
*Lignin-relevant trait*								
Lignin (% CWR^e^)	16.97	15.63	0	16.40 ± 0.81	14.63	18.21	4.94	0.06
H (mg g^−1^)	2.19	2.47	0.06	1.98 ± 0.39	1.08	2.68	19.70	0.08
S (mg g^−1^)	8.23	9.49	0.01	8.06 ± 1.66	4.29	11.47	20.60	0.08
G (mg g^−1^)	16.00	13.22	0.06	13.17 ± 3.28	5.73	21.00	24.91	0.08
H% (mol mol^−1^%)	9.04	10.95	0	9.71 ± 1.39	6.22	14.48	14.32	0.07
S% (mol mol^−1^%)	30.64	35.41	0	32.79 ± 2.38	26.71	38.33	7.26	0.06
G% (mol mol^−1^%)	60.32	53.64	0	57.51 ± 3.05	49.95	64.40	5.30	0.10
H/S (mol mol^−1^%)	29.52	30.92	0.26	29.69 ± 4.26	20.02	41.98	14.35	0.07
H/G (mol mol^−1^%)	14.98	20.41	0	17.01 ± 3.16	9.92	28.37	18.58	0.09
S/G (mol mol^−1^%)	50.82	66.04	0	57.37 ± 7.10	41.73	76.71	12.38	0.08
*Enzymatic digestibility*								
Xyl-Rel (% dry matter)	4.54	3.17	0.01	4.06 ± 1.39	1.79	8.58	34.24	0.13
Glc-Rel (% dry matter)	15.15	13.49	0.05	14.59 ± 2.20	9.92	20.92	15.08	0.08

^a^*P* value of *t*-test of the difference between two parents.

^b^Average ± standard deviation.

^c^Coefficient of variation (SD*100/mean).

^d^*P* value of Kolmogorov-Smirnov test.

^e^CWR, cell wall residue, represents the de-starched alcohol-insoluble residues.

**Figure 1:**
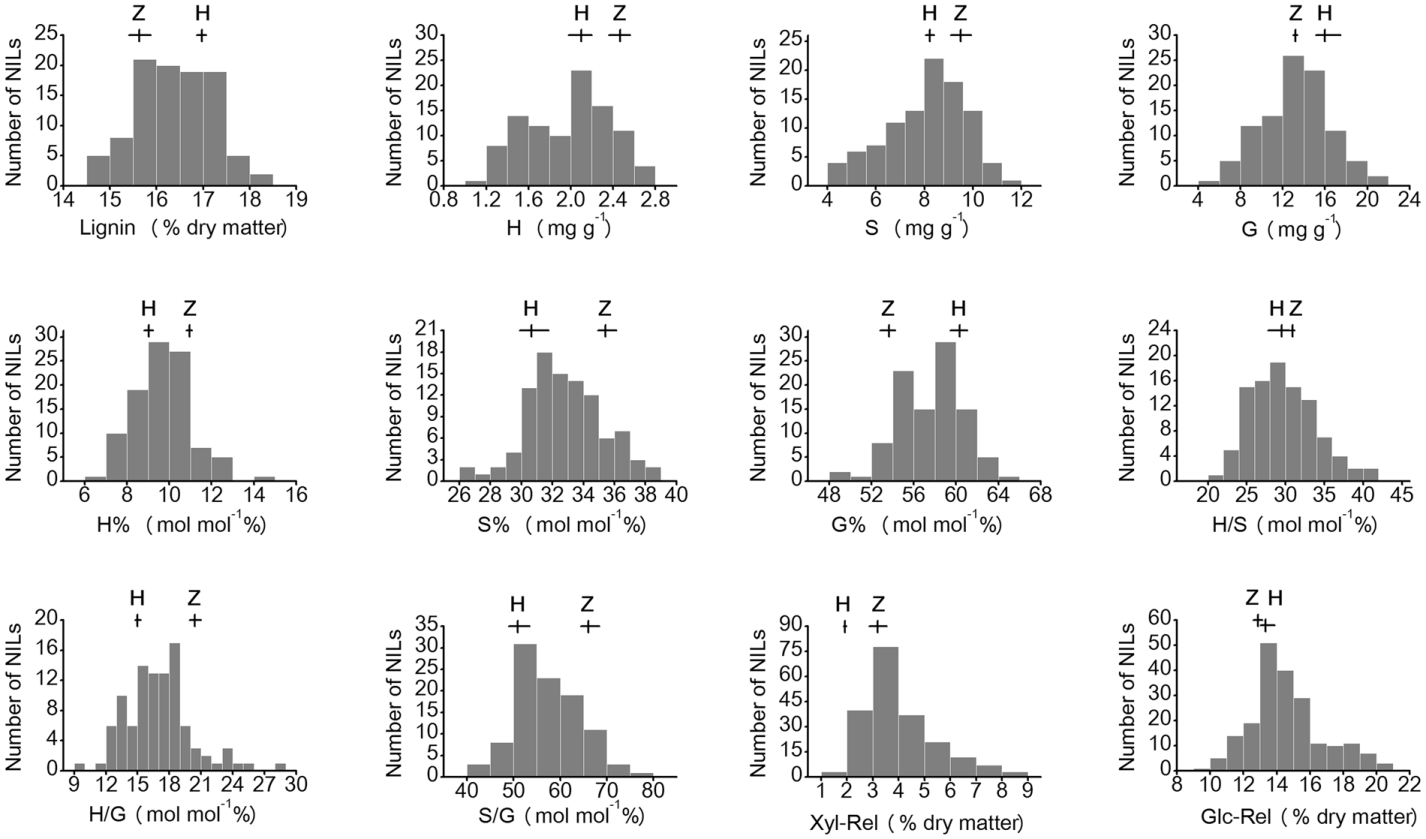
Frequency distributions of lignin-relevant traits and sugar releases in the RIL population. The values of two parents are indicated by “H” (Huahui-3) and “Z” (Zhongguoxiangdao) over the corresponding histogram. The short horizontal and vertical lines indicate the range and average value of two parents.

The three lignin monomers displayed considerable diversity with coefficient of variation (CV) were about 20%. Extensive variations among lines were also found for sugar release. By contrast, acetyl bromide lignin content exhibited low variation (CV < 5%) (Table 1). It indicated that the levels of lignin monomers could be quite different despite the acetyl bromide lignin content was similar in the rice lines. In addition, the transgressive segregation in both directions was found for both enzymatic digestibility and lignin monomers in the population (Fig. 1), which allowed us to select the favorable lines for further bioenergy crop breeding and to identify the multiple genetic factors controlling these traits.

### Lignin monomers rather than lignin content were correlated with biomass enzymatic digestibility after mild NaOH pretreatment

**Figure 2:**
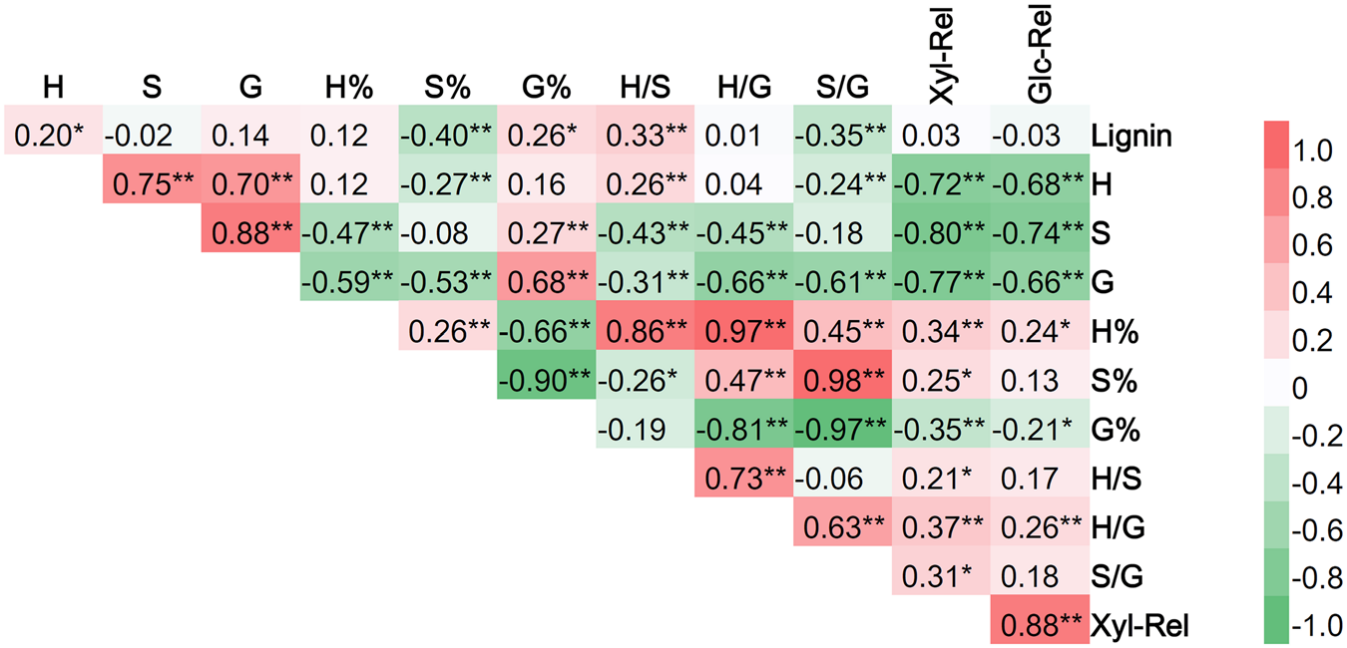
Correlations between lignin-relevant traits and sugar release. Red colors indicate positive correlations, while the green indicate negative correlations between traits. * and ** indicate that the correlations are significant at p < 0.05 and 0.01, respectively.

Lignin is considered a significant factor in recalcitrance to enzymatic hydrolysis. However, several recent findings have indicated that lignin content alone may not be the main factor that affects biomass digestibility^11,26^. In the present study, no correlation was found between acetyl bromide lignin content and sugar yields, whereas the levels of three lignin monomers all had significantly negative effects on Glc-Rel and Xyl-Rel (Fig. 2). In addition, the molar percentage of *p*-hydroxyphenyl unit (H%) and H/G were predicted to be beneficial for the sugar release of mature rice straw (Fig. 2).

### Identification of QTL for lignin monomers and biomass digestibility

Gaining genetic control of lignin-relevant traits and biomass digestibility is essential for optimizing straw characteristics and lignocellulose conversion. To map QTL for lignin content, lignin composition and biomass digestibility, a genetic map including 12 linkage groups and 181 markers was built, with a total genetic length of 1896.7 cM and average interval length of 11.1 cM (Supplementary Table S1). A total of 31 quantitative trait loci (QTL) were identified across six chromosomes for lignin-relevant traits and sugar release (Table 2, Supplementary Figure S1). Of them, 22 with LOD values above 2.0 were considered as significant QTL, while the other nine with the LOD values between 1.5–2.0 were considered as “suggestive QTL”. The total phenotypic variation (PV) explained by all the QTL for each trait varied from 8.11% for S% to 70.53% for S content. Sixteen QTL individually explaining more than 10% of PV were considered as major QTL and the others were considered as minor QTL (Table 2). The significant variation in lignin monomers allowed mapping of the relevant QTL. As expected, we mapped nine QTL associated with lignin monomers but no QTL for lignin content (Supplementary Figure S1). Two loci affecting absolute and relative values of lignin monomers were found, namely, the locus including *qS3* and *qS%*3 on chromosome 3 (denoted as chr.3) and the locus including *qH8* and *qH%8-1* on chr.8 (Supplementary Figure S1). However, the locus flanked by *RM495*-*RM4981* on chr.1 and the locus flanked by *RM3138*-*RM5463* on chr.6 only had effects on the absolute values of H and G, respectively. Interestingly, the QTL flanked by *RM585*-*RM276* on chr.6 affected the relative amounts of H and G in the opposite way, causing changes in H/G and S/G (Supplementary Figure S1). This may indicate that these QTL were the most likely candidates involved in different steps of monolignol biosynthesis or polymerization. Finally, three significant QTL with logarithm of odds (LOD) greater than 2.0 were identified for the Glc-Rel, located on chromosomes 6, 8 and 9 and contributed 6.39%, 10.43% and 7.4% of total variation (Table 2).

**Table 2:** Information of mapped QTL related to lignin-relevant traits and sugar release.

Traits	QTL	Chr.	Marker interval	Position (cM)	LOD	PV (%)^a^	Additive^b^
H	***qH1*** ^**c**^	1	*RM495-RM4981*	6	2.6	13.14	0.14
	***qH8***	8	*RM1376-RM310*	45	4.2	20.15	0.18
S	***qS3***	3	*RM520-RM422*	201	2.5	26.89	−0.88
	***qS7***	7	*RM3394-RM20916*	10	2.3	8.47	−0.48
	***qS8***	8	*RM1376-RM310*	46	6.8	35.17	1.00
G	***qG6***	6	*RM3138-RM5463*	197	2.1	8.16	−0.94
	***qG8-1***	8	*RM1376-RM310*	46	5.9	30.29	1.84
	***qG8-2***	8	*RM531-RM80*	108	2.5	12.20	−1.14
H%	***qH%1***	1	*RM8229-RM5*	151	2.1	9.27	−0.42
	***qH%6***	6	*RM585-RM276*	61	2.0	15.88	0.55
	*qH%7*	7	*RM20916-RM481*	20	1.5	7.52	0.38
	***qH%8-1***	8	*RM1376-RM310*	48	2.0	11.16	−0.47
	*qH%8-2*	8	*RM531-RM80*	105	1.7	9.03	0.42
S%	*qS%3*	3	*RM520-RM422*	184	1.8	8.11	−0.76
G%	***qG%6***	6	*RM585-RM276*	63	2.3	23.43	−1.47
H/S	*qH/S1*	1	*RM8229-RM5*	151	1.6	7.14	−1.13
	***qH/S7***	7	*RM20916-RM481*	18	2.0	11.37	1.43
H/G	***qH/G1***	1	*RM8229-RM5*	151	2.0	8.85	−0.94
	***qH/G6***	6	*RM585-RM276*	63	2.4	18.10	1.34
	***qH/G8-1***	8	*RM1376-RM310*	49	2.2	11.45	−1.08
	*qH/G8-2*	8	*RM531-RM80*	107	1.7	10.22	1.01
S/G	*qS/G6*	6	*RM585-RM276*	65	1.6	14.93	2.73
Xyl-Rel	*qXyl-Rel3*	3	*RM520-RM422*	199	1.5	6.81	0.37
	***qXyl-Rel8-1***	8	*RM1376-RM310*	47	10.0	24.54	−0.70
	***qXyl-Rel8-2***	8	*RM531-RM80*	103	2.1	4.07	0.28
	***qXyl-Rel9***	9	*RM434-RM242*	73	2.3	4.96	−0.31
Glc-Rel	*qGlc-Rel3*	3	*RM520-RM422*	195	1.8	7.97	0.64
	***qGlc-Rel6***	6	*RM3138-RM5463*	197	2.9	6.39	0.56
	***qGlc-Rel8-1***	8	*RM1376-RM310*	46	4.1	10.43	−0.72
	*qGlc-Rel8-2*	8	*RM531-RM80*	103	1.6	3.23	0.40
	***qGlc-Rel9***	9	*RM434-RM242*	68	2.6	7.40	−0.60

^a^Percentage of the trait variance explained by the QTL.

^b^Positive additive effect indicates the contribution derived from ZX and that from HH-3 is negative.

^c^The significant QTL with LOD value greater than 2.0 were bold.

Additive effects can show the contributions of two parents’ alleles to the final phenotypes. In this population, positive and negative additive effects indicated that the positive alleles came from ZX and HH-3, respectively. Consequently, the alleles for increasing phenotypic values were contributed by parent ZX at 16 loci, while at the remaining 15 loci, the positive alleles came from parent HH-3 (Table 2). It was shown in Table 2 that for the traits S content, G content, H%, H/S, Xyl-Rel and Glc-Rel, the positive alleles were dispersed in two parents. It’s can be a ready explanation for the transgressive segregation in both directions observed for both enzymatic digestibility and lignin monomers in the population. This result also indicated that ideal cell wall traits could result from the genetic combination of favorable alleles from the two parents.

### Co-localization of QTL indicated that lignin monomers and biomass digestibility were genetically correlated

**Figure 3:**
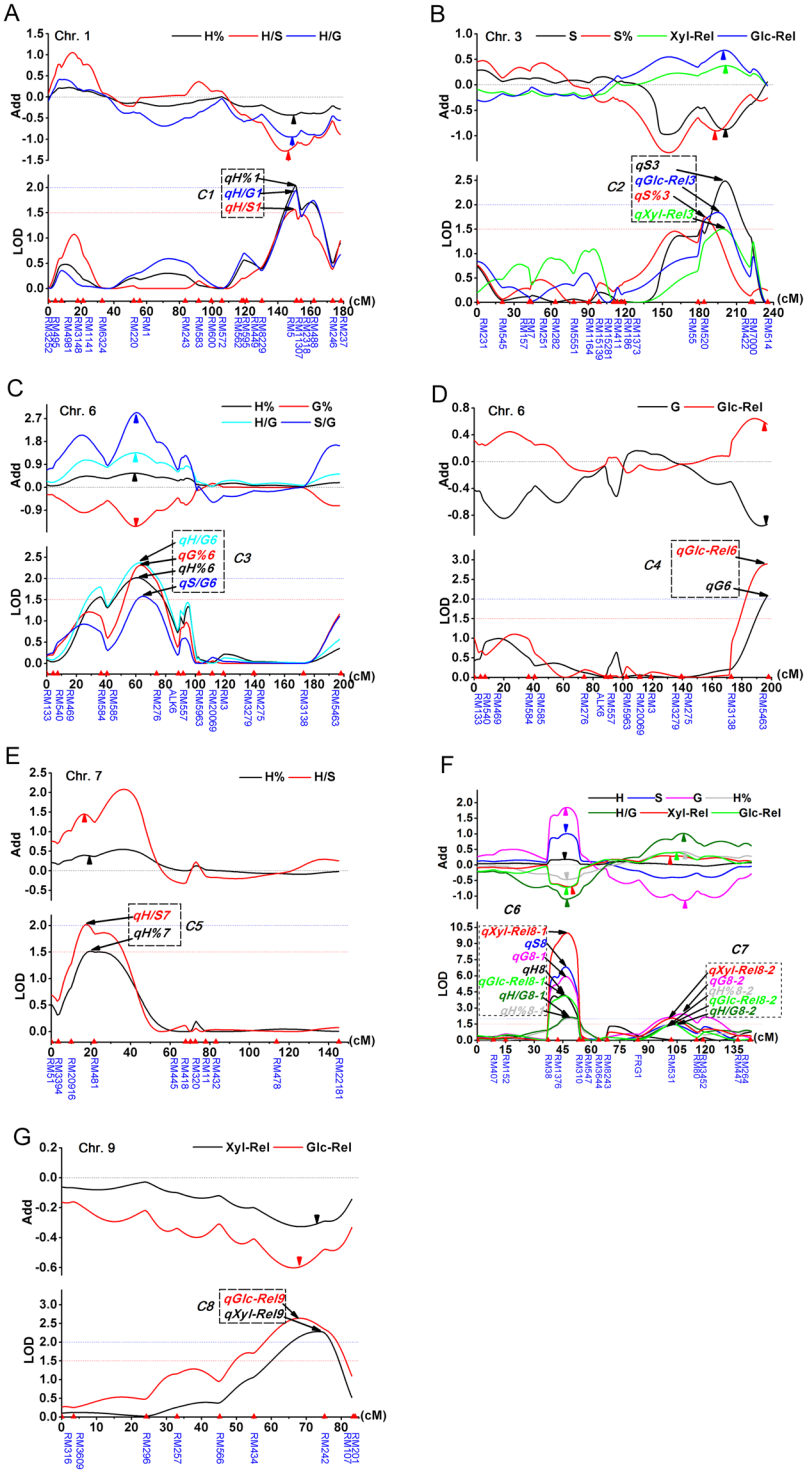
QTL identified and co-mapped for lignin monomers and sugar release. The positive and negative additive effects indicated that the positive alleles came from ZX and HH-3, respectively. The QTL clusters were framed with dotted lines.

We compared the location of the all mapped QTL including the “suggestive QTL” and found that the cell wall-relevant QTL were widely co-localized. In this process, we slightly lowered the threshold value to 1.5 with the purpose to find more minor QTL (“suggestive QTL”) for a comprehensive comparation. However, not all “suggestive QTL” will be included in clusters. Only if a minor QTL co-localized with at least a major one, it will be considered as a possible member QTL in a cluster. Twenty-nine main effect QTL were found to co-localize within the genome during the QTL cluster analysis and eight QTL clusters (*C1*-*C8*) were revealed and further classified into three groups, namely, clusters for lignin monomer-related traits (*C1, C3* and *C5*), clusters for multiple types of traits (affecting both lignin monomers and digestibility, *C2*, *C4*, *C6* and *C7*) and clusters for digestibility alone (*C8*) (Supplementary Figure S1, Fig. 3). For the three clusters related to lignin monomer related traits, *C1* on chr.1 and *C5* on chr.7 controlled the relative amounts of the H monomer (Fig. 3A,E). *C3* on the upper arm of chr.6 controlled the H% and G% with the allele from parent ZX decreased G% but increased H%, resulting in increased ratios of H/G and S/G (Fig. 3C).

Among the four QTL clusters for multiple traits, the allele from parent ZX at *C2* on chr.3 decreased S but increased the sugar release (Fig. 3B). The allele from parent ZX at *C4* on the lower arm of chr.6 decreased the G but increased Glc-Rel (Fig. 3D). The QTL cluster *C6* at the upper arm of chr.8 was largest, consisting of seven individual QTL members. The alleles from parent ZX at this locus increased all three monomers but simultaneously decreased the release of both glucose and xylose (Fig. 3F). The QTL cluster *C7* in the middle region of chr.8 was the second largest one. The alleles from parent ZX at this locus decreased the absolute amount of G but increased the H%, H/G, Glc-Rel and Xyl-Rel (Fig. 3F). Four of the eight QTL for lignin monomer abundance overlapped with those for saccharification yield, indicating that correlation of the lignin monomers with the biomass digestibility might be controlled by the same genetic factors. In general, QTL co-localizations were in good agreement with the observed correlation patterns among these traits. In addition, the last QTL cluster *C8* on chr.9 only significantly affected sugar release (Fig. 3G), indicating that genetic determinants for other cell wall characteristics other than lignin monomers influenced the recalcitrance.

To verify the co-localization of QTL, we further tried joint mapping approach to map the QTL associated multiple related traits using the QTL Cartographer software. Totally, 89 QTL and 9 QTL clusters were determined for lignin-relevant traits and biomass enzymatic digestibility (Supplementary Table 2). As examples, 8 QTL were determined by joint multi-trait analysis for Glc-Rel with correlative lignin-relevant traits. Three QTL on chromosomes 1, 3 and 8 were repeatedly mapped for Glc-Rel with H, S, G, H% and H/G. In addition, two QTL on chromosome 6 between *RM585-ALK6* and *RM3138-RM5463* were detected for Glc-Rel with H% and H/G. The joint mapping results were quite consistent with co-localization analysis. Furthermore, almost all of the co-localized QTL were exactly mapped in same interval, indicating that the possible pleiotropism of the QTL on cell wall-relevant traits.

### QTL information could be used to select favorite rice lines

**Figure 4:**
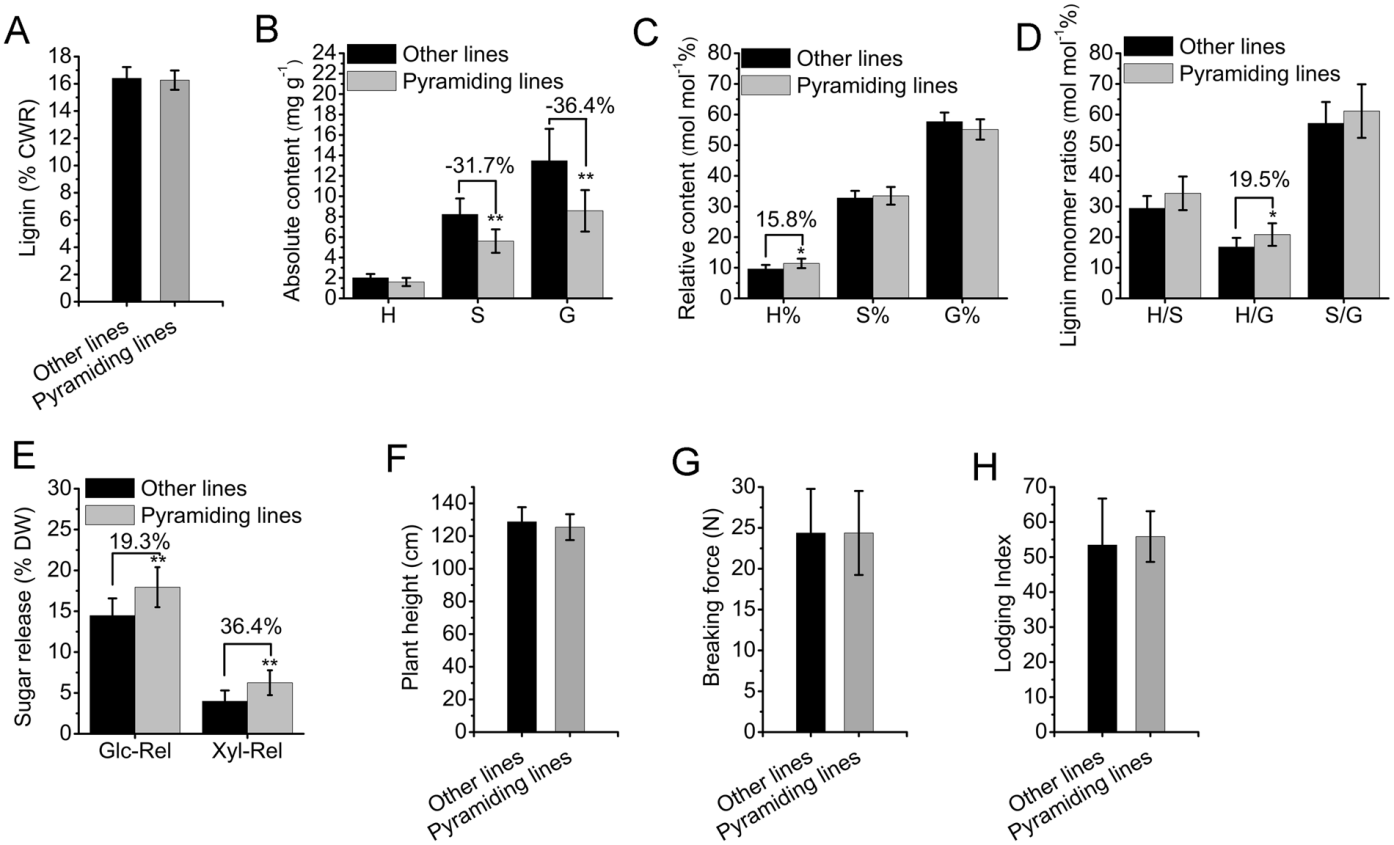
Comparing lignin-relevant traits, sugar releases, plant height, breaking force and lodging resistance of pyramiding lines (n = 8) and other lines (n = 91 in **A**–**D**,**F**–**H** and n = 207 in **E**). (**A**) Lignin content; (**B**) absolute content of lignin monomers; (**C**) molar percent of lignin monomers; (**D**) molar ratios between lignin monomers; (**E**) sugar releases; (**F**) plant height; (**G**) breaking force; (**H**) lodging index.

Since chemical methods for determining lignin content, lignin composition and biomass digestibility are normally time-consuming, the molecular markers closely linked to the QTL clusters simultaneously affecting lignin monomers and digestibility could be a convenient alternative for the selection of rice lines with modified lignin characteristics and improved biomass digestibility. As four markers, *RM520*, *RM5463*, *RM310* and *RM531*, were tightly linked to the four QTL clusters for multiple types of traits (Table 2), they were used to select pyramiding lines with modified linin composition and biomass digestibility. Thus, eight pyramiding lines with four positive alleles for increasing sugar release were selected (Supplementary Table S3). The pyramiding lines showed comparable lignin content but significantly decreased S or G compared to other lines (Fig. 4A,B). The H% and H/G increased 15.8% and 19.5% in pyramiding lines (Fig. 4C,D). Notably, the Glc-Rel and Xyl-Rel of pyramiding lines significantly increased by 19.3% and 36.4%, respectively (Fig. 4E).

The final objective of a breeding program is to obtain overall better performance of the variety. As lignin-related traits may influence the growth, culm strength and lodging resistance of rice^26,27^, we investigated the plant height, breaking force and lodging index of pyramiding lines and other lines to investigate whether modified lignin characteristics and improved biomass digestibility will damage these traits in rice. There were no significant differences between the pyramiding lines and other lines in terms of the above traits (Fig. 4F,G,H). We further compared grain shape, 1000-seed weight and grain eating/cooking qualities in pyramiding lines and other lines using data published by Yan *et al*.^28,29^. The *t*-test showed no significant difference between pyramiding lines and other lines in most traits, except for slightly decreased gel consistency (GC) or increased final viscosity at 40 °C (FV) in pyramiding lines (Supplementary Table S4). In conclusion, modification of lignin characteristics and improvement of sugar release by combining the four positive alleles indicated by QTL information will not sacrifice the normal growth or grain quality of rice.

## Discussion

Extensive evidence has suggested that lignin content and composition greatly influence the effective fractionation of plant biomass into fermentable sugars^13,30^. Gaining genetic control of lignin composition is essential for optimizing lignocellulose conversion and straw characteristics without compromising plant vigor and biomass yield. Here, we analyzed the lignin content, lignin composition and biomass digestibility of rice straw samples from a RIL population composed of different genotypes using a high throughput platform, which allowed us to correlate biomass enzymatic digestibility with various lignin properties at the genetic level. As a result, H% and H/G showed a positive correlation with biomass digestibility while lignin monomers, rather than acetyl bromide lignin, had negative effect on sugar release. The pyramiding rice lines selected by QTL information displayed decreased S or G and increased H%, H/G and biomass digestibility as well as normal agronomic traits, which suggested a convenient method for selecting outstanding rice lines at the population scale and a feasible direction for modifying lignin-relevant traits.

The impacts of lignin composition on biomass recalcitrance have been inconsistent in previous research. Abundant evidence has shown that lignin is a major deterrent to enzyme attack on cellulose and thus it negatively influences biomass digestibility^7,31^. Several reports, however, have indicated that lignin may not always be a negative factor for saccharification efficiency^10,11^. The inconsistent results are mainly due to complications arising from remarkable variances in plant type, the origin and maturity of plant materials, cell wall composition and the selection of a pretreatment method^32^. In this study, acetyl bromide lignin content had no significant correlation with sugar release (Figure S1C), even when the mild conditions (6.25 mM NaOH at 90 °C for 3 h) were used for pretreatment to avoid melting or partially removing lignin from the cell wall, which can be induced by strong pretreatment^33^. One of the reasons is that the lignin monomers extracted mostly represent the “active” or “branched” lignin that tightly crosslinked with polysaccharides thus affecting sugar release, whereas the total lignin content mainly represent “fixed” lignin that has less effects on cell wall recalcitrance. Many previous studies reported that genetic engineering of the lignin monomer biosynthetic genes by down or up-regulation of the genes such as *PAL*, *C4H*, *HCT*, *F5H*, *COMT*, *CCoMAT and CAD*, resulted in the changes of H, S/G, 5-OH-G, thus had effects on forage digestibility^34^. Another reason for this uncorrelated relationship may be the small variation in lignin content (CV less than 5%) in this population, as the influence of lignin on biomass digestibility might be masked by other cell wall characteristics. In contrast, lignin monomer levels displayed considerable variation (CV approximately 20%), which provided a chance to explore how to modify lignin composition without changing lignin content. Notably, the pyramiding lines with modified lignin composition and increased biomass digestibility displayed normal plant height, lodging resistance, grain shape and weight and cooking/eating qualities, which might also be ascribed to the normal lignin content. The identified QTL with divergent effects on the monomer ratios and/or the sugar release will be beneficial in later genetic modifications of lignin composition to improve the biomass digestibility.

While the absolute contents of all three kinds of lignin monomers played negative roles on biomass digestibility, H%, S% and G% displayed diverse influences on sugar release. H% was a positive factor; S% had no correlation with Glc-Rel and G% was contrary to H%. Consequently, H/G was also a positive factor. These correlations were the same as in the previous research, in which the cleavage of β-O-4 linkages in the H-lignin dominant mutant was higher than in the G-lignin dominant mutants^32^. We speculated that G monomer, which could covalently crosslink with up to three other monomers, was more internal than S and H in the lignin network. As the correlation between lignin monomers and digestibility has been shown previously, it is reasonable to find the QTL associated with both of them.

The multiple effects of lignin-monomer related QTL indicated that the pathways for different monomers can be regulated independently. The QTL clusters on chromosomes 1 and 7 only had a detectable effect on H; the QTL cluster on the lower arm of chromosome 6 only had a significant effect on G; the QTL cluster on the upper arm of chromosome 6 had an opposite effect on S and G; the QTL cluster in the middle region of chromosome 8 had the same effect on both S and G and the QTL cluster on the upper arm of chromosome 8 had similar effects on three monomers (Fig. 3). In addition to the generally coordinated control of monolignol biosynthesis in secondary growth, these genetic loci exemplified the complex events of lignin biosynthesis.

A major hindrance to the development of high-yielding biofuel feedstocks is the ability to rapidly assess large populations of plants for fermentable sugar yields. Here, we present a holistic high-throughput methodology for assessing rice straws harvested at the mature stage, including lignin content, lignin monomers and fermentable sugar yields. To our knowledge, this is the first time to demonstrate that a rice RIL population could be used to screen favorite lines and explore the genetic basis underlying lignin characteristics and biomass digestibility. The identification of a considerable number of QTL clearly confirmed that the high-throughput method was reliable.

Although QTL information could be a convenient alternative for the selection of well-preformed lines with modified lignin composition and improved biomass digestibility, efforts to breed desirable energy plants should consider the following factors: (1) Since cell wall characteristics including cellulose crystallinity and cross-linking of polysaccharides can also influence biomass recalcitrance^35–37^, the QTL cluster mapped in this study might also have some overlap with QTL for polysaccharides. Therefore, further QTL mapping should be performed for polysaccharides to gain a comprehensive understanding of biomass characteristics. (2) Epistasis may play an important role on genetic control of lignin-relevant traits and biomass digestibility. In this study, we found the QTL (*RM1376-RM310*) might be interactive with two other loci (*RM520-RM422* & *RM258-RM591*) for the trait Xyl-Rel. Larger populations and higher density genetic maps are required in further studies to detect the epistasis effects of the QTL. (3) A pervasive problem in QTL studies is the difficulty in identifying causative genes within large physical intervals as in this study (Supplementary Table S5). So, the next step is to further narrow the QTL region and thus this study can serve as a basis for further functional studies.

## Materials and Methods

### Experimental materials

As described previously with minor changes^28^, the population with 215 RILs was developed from a cross between the rice cultivars ‘Huahui3’ (HH-3) and ‘Zhongguoxiangdao’ (ZX) through the single seed descent method. HH-3 is an improved version of ‘Minghui 63’ with a bacterial blight resistance gene (*Xa21*) and a lepidopteran insect resistance gene (*Bt*). ZX is an aromatic rice variety with high grain quality that is largely grown in south China. The population and parents were planted in the experimental station of Huazhong Agricultural University (Wuhan, China) during the 2012 growing season. Ten individuals from each line were grown in single rows with a distance of 17 cm between plants in each row and 27 cm between rows. At the mature stage, three to five biological replicates from each line were pooled. The leaves of postharvest rice straw were removed and the stems were dried at 60 °C to a constant weight. The dried stem tissues were ground and the powders were sieved through a 40 mesh sieve and retained for analysis, with at least three technical replicates for all experiments.

### Enzymatic saccharification assay

The high-throughput digestibility platform used for chemical pretreatment and subsequent enzymatic hydrolysis of residues has been described previously^38^. The automated grinding, feeding and weighing of dried rice material was performed by a custom-designed robot known as iWALL. Samples of dried plant material (1.5 mg) were pretreated with 6.25 mM NaOH at 90 °C for 3 h in a water bath. Neutralization and saccharification were performed with 0.25 μL Accellerase 1000 (Genencor, Rochester, NY) in 30 mM citrate buffer (pH 4.5) plus 0.01% sodium azide. Glucose and xylose release were determined with enzyme-based colorimetric assays (Megazyme International Ireland, Wicklow, Ireland). Glucose concentrations were assayed with the glucose oxidase/peroxidase (GOPOD) method (K-GLUC, Megazyme, Ireland) using 4 μL of the supernatant of the digestion reaction mixture and 64 μL of the GOPOD assay reagent. Xylose concentrations were assayed enzymatically (K-XYLOSE, Megazyme) using 8 μL sample and 62 μL assay reagent.

### Cell wall extraction and acetyl bromide assay of lignin

The methods for cell wall isolation, lignin content and lignin composition were previously described^39^. Approximately 60–70 mg of dried plant material was ground and dispensed by iWALL. The de-starched alcohol-insoluble residues representing the isolated cell wall material were prepared by washing each sample with 1.5 ml of 70% aqueous ethanol and 1.5 ml of chloroform/methanol (1:1, v/v), followed by treatment with 35 μl of 0.01% sodium azide (NaN_3_), 35 μl of amylase (50 μg/mL in H_2_O, from Bacillus species, Sigma) and 17 μl pullulanase (17.8 units, from Bacillus acidopullulyticus, Sigma). The isolated cell wall material (1–1.5 mg) was transferred into a 2 ml volumetric flask leaving one tube empty for a blank; then 100 μl of freshly made acetyl bromide solution (25 ml acetyl bromide in 100 ml glacial acetic acid) was gently added. The flask was incubated at 50 °C for 2 h and then heated for an additional hour with vortexing every 15 minutes. After cooling on ice to room temperature, sodium hydroxide (400 μl of 2 M) and 70 μl of freshly prepared 0.5 M hydroxylamine hydrochloride were added. The flask was filled up to the 2.0 ml mark with glacial acetic acid and then capped and mixed by inverting several times. The solution (200 μl) was pipetted into a UV-transparent 96 well plate and the absorption was read using a Shimadzu UV-1800 spectrophotometer at 280 nm. The percentage of acetyl bromide soluble lignin (% ABSL) was determined using the equation (1). For grasses, a coefficient of 17.75 was used.

1 $$ \% \,{\rm{ABSL}}=\frac{{\rm{absorbance}}\times {\rm{total}}\,{\rm{volume}}\times \mathrm{100} \% }{{\rm{coefficient}}\times {\rm{path}}\,{\rm{length}}\times {\rm{weight}}}$$

### Lignin composition assay

The isolated cell wall material (2 mg) was transferred into a screw-capped glass tube for thioacidolysis. Dioxane (175 μl), ethanethiol (EtSH, 20 μl 10%) and boron trifluoride diethyl etherate (BF_3_, 5 μl 2.5%) were added. The suspension was heated at 100 °C for 4 h with gentle mixing every hour following by cooling on ice for 5 min. Sodium bicarbonate (150 μl of 0.4 M) was added to the sample and vortexed. Water (1 ml) and ethyl acetate (0.5 ml) were added and vortexed. The top layer (ethyl acetate) (150 μl) was transferred to a 2 ml Sarstedt tube. The solvent was evaporated by a concentrator with air. For the TMS derivatization, 500 μl of ethyl acetate, 20 μl of pyridine and 100 μl of N, O-bis (trimethylsilyl) acetamide was added together and the mixture was incubated for 2 h at 25 °C. The reaction (100 μl) was transferred to a GC/MS vial and 100 μl of acetone was added. The sample was analyzed by GC equipped with a quadrupole mass-spectrometer or flame ionization detector. An Agilent HP-5 MS column was installed (30 × 0.25 mm × 0.25 μm film thickness). The following temperature gradient was used with a 30 min solvent delay and a 1.1 ml/min flow rate, initial held at 130 °C for 3 min, a 3 °C/min ramp to a 250 °C and held for 1 min, allowed equilibration to the initial temperature of 130 °C. Peaks were identified by characteristic mass spectrum ions of 299 m/z, 269 m/z and 239 m/z for S, G and H monomers, respectively. The composition of the lignin components was quantified by setting the total peak area to 100%. The contents of H, S and G were calculated as the qualities of the monomers (mg) to the total qualities of the cell wall residue (g). The H%, S% and G% were calculated as the mole percentage of the monomers to the total monomers (H + S + G).

### Linkage map construction and QTL mapping

A total of 177 simple sequence repeat (SSR) markers and four insertion deletion (InDel) markers were employed to construct the genetic map of the RIL population using MAPMAKER/EXP version 3.0b software^40^, in which 145 SSR and all InDel markers have been described previously^28^. In this study, an additional 32 markers were added to the map using the SSR assay as described^41^. A total of 12 linkage groups were produced with a minimum logarithm of odds (LOD) score of 3.0. Genetic distances were calculated with the Kosambi map function. Inclusive composite interval mapping was carried out using QTL IciMapping version 3.3 based on stepwise regression^42^, with simultaneous consideration of all marker information. The walking speed chosen for all QTL was 1.0 cM. For all traits analyzed, a LOD threshold of 2.0 was sufficient to detect a significant QTL and a LOD threshold of 1.5 was sufficient to detect a suggestive QTL^43^. Epistasis effects between two loci were estimated also by QTL IciMapping version 3.3. In a stepwise regression, mapping parameters were set as 5 cM steps and 0.001 probabilities and the LOD threshold was set to 5. In addition, a multiple-trait version of composite interval mapping was conducted using the QTL Cartographer version 2.5 software^44^, to test the genetic relationships between sugar releases and lignin-relevant traits.

### Plant height, breaking force and lodging index measurement

The plant height, breaking force and lodging index of the second internode (from base to top) were measured at 30 days after flowering in the 2013 growing season. Ten primary tillers from 20 plants were selected for each line. The length from the base of culm to the apex of panicle represented plant height. The breaking force (BF) was measured using a prostrate tester (DIK 7400, Japan) with the distance between two fulcra set as 5 cm. The breaking site was arranged at the center of the second internode and the middle point between two fulcra. The lodging resistance of the second internode was measure as described previously^45^. The length (L) and fresh weight (W) of the section from the panicle to the middle of the second internodes were measured. Lodging index (LI) of the second internode was calculated using the equation (2). The date of ten tillers was averaged before used to further analysis.

2 $${\rm{LI}}=\frac{{\rm{L}}\times {\rm{W}}\times 9.8}{{\rm{BF}}\times 5\times 1000}\times {\rm{100}}$$

### Statistical analysis

The Pearson correlation analysis for all traits based on the mean values, the *t*-test and the Kolmogorov-Smirnov test were performed using statistics software of the SPSS 17.0 (SPSS, Chicago, IL, USA; http://en.wikipedia.org/wiki/SPSS).

### Data availability statement

All data generated or analyzed in this study are included in this published article and its Supplementary Information files.

## Electronic supplementary material

Supplementary imformation
