# Supplementary material for: Genetic loci simultaneously controlling lignin monomers and biomass digestibility of rice straw
Source: Sci Rep. 2018 Feb 26;8:3636. doi: 10.1038/s41598-018-21741-y (PMC5827516; doi:10.1038/s41598-018-21741-y)
Supplement: Supplementary file 1 — Supplementary imformation [file 41598_2018_21741_MOESM1_ESM.pdf]

Supporting Information

Genetic loci simultaneously controlling lignin monomers and biomass digestibility of rice straw

Zhen Hu[1,2,3], Guifen Zhang[1,2,3], Ali Muhammad[1,2,3], Rana Abdul Samad[1,2,3], Youmei Wang[1,2,3], Jonathan D. Walton[4], Yuqing He[2,5], Liangcai Peng[1,2,3], and Lingqiang Wang[1,2,3]*

[1]Biomass and Bioenergy Research Centre, Huazhong Agricultural University, Wuhan, China

[2]National Key Laboratory of Crop Genetic Improvement, Huazhong Agricultural University, Wuhan, China

[3]College of Plant Science and Technology, Huazhong Agricultural University, Wuhan, China

[4]Department of Energy Plant Research Laboratory and DOE Great Lakes Bioenergy Research Center, Michigan State University, East Lansing, MI 48824, USA

[5]College of Life Science and Technology, Huazhong Agricultural University, Wuhan, China

*Corresponding author: Lingqiang Wang (lqwang@mail.hzau.edu.cn). Huazhong Agricultural University, Wuhan 430070, China. Tel.: +86 27 87281765; fax: +86 27 87280016.

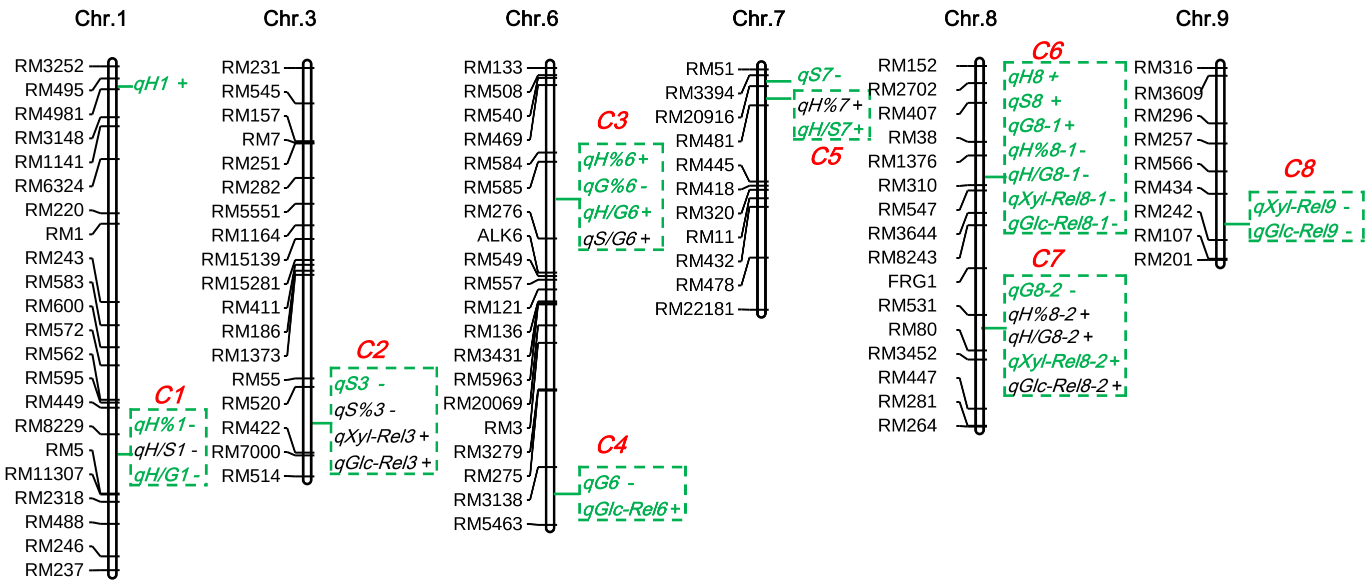

Supplementary Figure S1. Distribution of the QTL for lignin monomers and sugar release on the linkage maps. The marker names were shown on the left of the chromosomes, while the QTL were displayed on the right of the chromosomes. The QTL co-localized in the same regions were framed. The "+" and "-" indicated that the positive alleles came from ZX and HH-3, respectively. The green font means the LOD value of QTL is greater than 2.0 and the black font means the LOD value is greater than 1.5 but less than 2.0.

Supplementary Table S1. Imformation of genetic map.

Linkage group	Number of markers	Length of linkage group (cM)	Average distance (cM)
Chr.1	22	178.4	8.1
Chr.2	21	201.2	9.6
Chr.3	18	235.3	13.1
Chr.4	21	219.1	10.4
Chr.5	19	172.2	9.1
Chr.6	20	197.9	9.9
Chr.7	11	145.1	13.2
Chr.8	16	134.8	8.4
Chr.9	9	83.9	9.3
Chr.10	7	141.4	20.2
Chr.11	10	119.4	11.9
Chr.12	7	68.0	9.7
Total	181	1896.7	11.1

Supplementary Table 2. Putative QTLs determined by joint analysis for H with H%, S with S%, G with G%, H with S, H with G, S with G, sugar releases with lignin-relevant traits and Xyl-Rel with Glc-Rel.

Multiple traits	Chromosome	Marker interval	Position (cM)	LOD
H with H%	1	*RM3252-RM3148*	6	3.5
S with S%	1	*RM3252-RM3148*	2	3.9
G with G%	1	*RM3252-RM3148*	4	3.2
H with S	1	*RM3252-RM3148*	6	4.4
H with G	1	*RM3252-RM3148*	6	4.3
H with Xyl-Rel	1	*RM3252-RM3148*	0	4.4
S with Xyl-Rel	1	*RM3252-RM3148*	0	7.3
G with Xyl-Rel	1	*RM3252-RM3148*	0	5.1
H% with Xyl-Rel	1	*RM3252-RM3148*	2	3.9
S% with Xyl-Rel	1	*RM3252-RM3148*	0	3.2
H/S with Xyl-Rel	1	*RM3252-RM3148*	12	3.6
H/G with Xyl-Rel	1	*RM3252-RM3148*	2	3.6
S/G with Xyl-Rel	1	*RM3252-RM3148*	0	2.9
H with Glc-Rel	1	*RM3252-RM3148*	2	4.2
S with Glc-Rel	1	*RM3252-RM3148*	0	6.5
G with Glc-Rel	1	*RM3252-RM3148*	0	4.7
H% with Glc-Rel	1	*RM3252-RM3148*	0	4.1
S% with Glc-Rel	1	*RM3252-RM3148*	2	3.6
G% with Glc-Rel	1	*RM3252-RM3148*	2	2.8
H/S with Glc-Rel	1	*RM3252-RM3148*	2	2.8
H/S with Glc-Rel	1	*RM3252-RM3148*	10	3.4
H/G with Glc-Rel	1	*RM3252-RM3148*	4	3.7
S/G with Glc-Rel	1	*RM3252-RM3148*	2	3.1
Xyl-Rel with Glc-Rel	1	*RM3252-RM3148*	0	2.9
S with S%	1	*RM1-RM583*	70	2.6
S/G with Xyl-Rel	1	*RM1-RM583*	74	2.8
S/G with Glc-Rel	1	*RM1-RM583*	86	2.7
H% with Xyl-Rel	1	*RM11307-RM488*	151	3
H/S with Xyl-Rel	1	*RM11307-RM488*	156	4
H/G with Xyl-Rel	1	*RM11307-RM488*	151	2.7
H/S with Glc-Rel	1	*RM11307-RM488*	156	3.7
G with G%	3	*RM231-RM545*	0	3.8
S/G with Glc-Rel	3	*RM231-RM545*	0	2.5
S with S%	3	*RM186-RM1373*	163	3.4
G with G%	3	*RM186-RM1373*	157	3.5
G with Xyl-Rel	3	*RM520-RM422*	184	2.9
S% with Xyl-Rel	3	*RM520-RM422*	186	3.9
G% with Xyl-Rel	3	*RM520-RM422*	184	4.7
H/G with Xyl-Rel	3	*RM520-RM422*	184	3.1
S/G with Xyl-Rel	3	*RM520-RM422*	184	4
H with Glc-Rel	3	*RM520-RM422*	190	2.9
S with Glc-Rel	3	*RM520-RM422*	190	2.7
G with Glc-Rel	3	*RM520-RM422*	186	3.1
H% with Glc-Rel	3	*RM520-RM422*	190	3
S% with Glc-Rel	3	*RM520-RM422*	184	4.2
G% with Glc-Rel	3	*RM520-RM422*	184	5.1
H/S with Glc-Rel	3	*RM520-RM422*	186	2.6
H/G with Glc-Rel	3	*RM520-RM422*	184	3.6
S/G with Glc-Rel	3	*RM520-RM425*	184	4.2

Supplementary Table 2 continued

Multiple traits	Chromosome	Marker interval	Position(cM)	LOD
H% with Xyl-Rel	5	*MRG2228-RM413*	28	2.5
G with G%	6	*RM585-ALK6*	73	2.6
H% with Xyl-Rel	6	*RM585-ALK6*	82	3.2
G% with Xyl-Rel	6	*RM585-ALK6*	65	2.5
H/G with Xyl-Rel	6	*RM585-ALK6*	71	3.2
H% with Glc-Rel	6	*RM585-ALK6*	82	3.3
G% with Glc-Rel	6	*RM585-ALK6*	65	2.5
H/G with Glc-Rel	6	*RM585-ALK6*	74	3.3
H% with Xyl-Rel	6	*RM557-RM121*	94	2.9
H% with Glc-Rel	6	*RM3138-RM5463*	189	2.7
H/S with Glc-Rel	6	*RM3138-RM5463*	191	3
H/G with Glc-Rel	6	*RM3138-RM5463*	197	3
Xyl-Rel with Glc-Rel	6	*RM3138-RM5463*	187	3.3
H with H%	8	*RM1376-RM310*	37	8.2
S with S%	8	*RM1376-RM310*	37	8.6
G with G%	8	*RM1376-RM310*	39	9
H with S	8	*RM1376-RM310*	37	8.4
H with G	8	*RM1376-RM310*	39	8.3
H with Xyl-Rel	8	*RM1376-RM310*	37	5.5
S with Xyl-Rel	8	*RM1376-RM310*	37	8.4
G with Xyl-Rel	8	*RM1376-RM310*	39	8.4
H% with Xyl-Rel	8	*RM1376-RM310*	37	5.6
S% with Xyl-Rel	8	*RM1376-RM310*	37	4.8
G% with Xyl-Rel	8	*RM1376-RM310*	37	5
H/S with Xyl-Rel	8	*RM1376-RM310*	37	5.7
H/G with Xyl-Rel	8	*RM1376-RM310*	39	6.9
S/G with Xyl-Rel	8	*RM1376-RM310*	37	4.8
H with Glc-Rel	8	*RM1376-RM310*	37	4.6
S with Glc-Rel	8	*RM1376-RM310*	37	8.6
G with Glc-Rel	8	*RM1376-RM310*	39	8.3
H% with Glc-Rel	8	*RM1376-RM310*	39	4.4
S% with Glc-Rel	8	*RM1376-RM310*	37	3.2
G% with Glc-Rel	8	*RM1376-RM310*	37	3.7
H/S with Glc-Rel	8	*RM1376-RM310*	37	4.1
H/G with Glc-Rel	8	*RM1376-RM310*	39	5.9
S/G with Glc-Rel	8	*RM1376-RM310*	37	3.4
Xyl-Rel with Glc-Rel	8	*RM1376-RM310*	39	12.1
G with G%	8	*RM8243-FRG1*	59	2.8
Xyl-Rel with Glc-Rel	9	*RM434-RM242*	71	3.3
Xyl-Rel with Glc-Rel	10	*RM25003-BT*	24	2.7

Supplementary Table S3. The QTL information used to selecting pyramiding lines with four positive alleles for improving sugar release.

	RM520	RM5463	RM310	RM531
Information of linked QTL cluster				
Name of QTL cluster	*C2*	*C4*	*C6*	*C7*
Additive effect of QTL of sugar releases [a]	+	+	-	+
Genotype of pyramiding lines [b]				
w7	2	2	0	2
w20	2	2	0	2
w53	2	2	0	2
w54	2	2	0	2
w63	2	2	0	2
w102	2	2	0	2
w125	2	2	0	2
w200	2	2	0	2

[a] "+" means the positive allele for improving sugar releases come from ZX and "-" means the positive allele came from HH-3.

[b] The genotype of rice lines same as ZX was recorded as "2", in opposite, was recorded as "0".

Supplementary Table S4. Comparisons of agronomic and eating/cooking quality traits between pyramiding lines and other lines.

Traits	Other lines (n=207)	Pyramiding lines (n=8)	*P* value of *t*-test
Grain shape and yield			
Grain length (mm)	9.7 ± 0.4	9.7 ± 0.5	0.84
Grain width (mm)	2.8 ± 0.1	2.7 ± 0.1	0.12
Grain thickness (mm)	2 ± 0.1	1.9 ± 0.1	0.33
1000-grain weight (g)	2.6 ± 0.3	2.6 ± 0.3	0.81
Eating and cooking quality			
ASV	3.1 ± 1.5	2.4 ± 1.4	0.29
AC (%)	12.7 ± 1.9	13.7 ± 4.8	0.58
GC (mm)	78.6 ± 7.9	72.3 ± 5.2	0.02
A Time (min)	6.2 ± 0.7	6.6 ± 0.7	0.31
B Time (min)	7.9 ± 0.1	7.9 ± 0.1	0.71
BA Time (min)	1.6 ± 0.7	1.3 ± 0.7	0.30
A Temp (℃)	76.8 ± 4.2	79 ± 4	0.26
B Temp (℃)	87.9 ± 1	87.9 ± 0.9	0.93
BA Temp (℃)	11 ± 4.2	8.9 ± 4.1	0.28
VA (BU)	25.2 ± 7	27.7 ± 9.4	0.54
PKV (BU)	432.2 ± 33.2	429.2 ± 10.5	0.55
V95 (BU)	336.3 ± 24.8	343.8 ± 25.4	0.51
HPV (BU)	233.5 ± 35.4	239.3 ± 37.8	0.73
CPV (BU)	288.1 ± 25.2	297.8 ± 17.6	0.24
FV (BU)	265.6 ± 24.6	278.8 ± 12.2	0.04
BDV (BU)	198.6 ± 37.2	188.3 ± 32.8	0.48
CS (BU)	52.5 ± 23.9	55.8 ± 25.7	0.77
SB (BU)	-145.2 ± 33.2	-131.3 ± 16.8	0.10

ASV, Alkali spreading value. AC, Amylose content. GC, Gel consistency. A Time, Pasting time. B Time, Peak time. BA Time, Time needed from initial viscosity increase to peak viscosity. A Temp, Pasting temperature. B Temp, Peak temperature. BA Temp, Temperature needed from initial viscosity increase to peak viscosity. VA, Pasting viscosity. PKV, Peak viscosity. V95, Viscosity at 95℃. HPV, Hot paste viscosity. CPV, Cool paste viscosity. FV, Final viscosity at 40 ℃. BDV, Breakdown viscosity. CS, Consistency viscosity. SB, Setback viscosity. BU, Brabender units.

Supplementary Table S5. Physical intervals of QTL.

QTL	Marker interval	Physical intervals (Kb)
qH1	*RM495-RM4981*	418
qH8	*RM1376-RM310*	613
qS3	*RM520-RM422*	2784
qS7	*RM3394-RM20916*	1179
qS8	*RM1376-RM310*	613
qG6	*RM3138-RM5463*	2384
qG8-1	*RM531-RM80*	2009
qG8-2	*RM1376-RM310*	613
qH%1	*RM8229-RM5*	5787
qH%6	*RM585-RM276*	3074
qH%8	*RM1376-RM310*	613
qG%6	*RM585-RM276*	3074
qH/S7	*RM20916-RM481*	1070
qH/G1	*RM8229-RM5*	5787
qH/G6	*RM585-RM276*	3074
qH/G8	*RM1376-RM310*	613
qXyl-Rel8-1	*RM531-RM80*	2009
qXyl-Rel8-2	*RM1376-RM310*	613
qXyl-Rel9	*RM434-RM242*	3033
qGlc-Rel6	*RM3138-RM5463*	2384
qGlc-Rel8	*RM1376-RM310*	613
qGlc-Rel9	*RM434-RM242*	3033
